# Analysis of the predictive role and new proposal for surgical strategies based on the modified Tomita and Tokuhashi scoring systems for spinal metastasis

**DOI:** 10.1186/1477-7819-12-245

**Published:** 2014-08-01

**Authors:** Junhyung Kim, Sun-Ho Lee, Se-Jun Park, Sung-Soo Chung, Eun-Sang Kim, Whan Eoh, Chong-Suh Lee

**Affiliations:** 1 Department of Neurosurgery, Samsung Medical Center, Sungkyunkwan University School of Medicine, 50 Irwon-dong, 135-710 Seoul, Gangnam-gu, South Korea; 2 Department of Orthopedic Surgery, Samsung Medical Center, Sungkyunkwan University School of Medicine, 50 Irwon-dong, 135-710 Seoul, Gangnam-gu, South Korea; 3 Spine Center, Samsung Medical Center, Sungkyunkwan University School of Medicine, 50 Irwon-dong, 135-710 Seoul, Gangnam-gu, South Korea

**Keywords:** Metastasis, Score, Spine, Survival, Validity

## Abstract

**Background:**

We sought to identify preoperative factors significantly correlated with survival. We also aimed to evaluate the validity of the prognostic scores in the Tomita and Tokuhashi systems and discuss several aspects to improve the predictive accuracy of these systems. Moreover, we suggest modified criteria for selecting treatment strategies.

**Methods:**

In total, the outcomes of 112 patients with spinal metastasis who underwent surgery between January 2006 and June 2011 were retrospectively reviewed. The validity of the prognostic scores was assessed on the basis of their correlation with survival. For various primary malignancies, new scoring criteria were applied in each system according to the survival results obtained in this study. Each revised scoring system was adjusted with a similar principle of scoring as described previously. Patient survival according to each preoperative factor was analyzed by the Kaplan-Meier method. The predictive value of each scoring system was evaluated by the log-rank test and Cox regression analysis.

**Results:**

The interval from the diagnosis of the primary malignancy to that of spinal metastasis (p = 0.023) and the interval from the diagnosis of spinal metastasis to surgery (p = 0.039) were significantly correlated with survival. Regarding Tokuhashi scores, the correlation coefficient was 0.790 before adjustment (p = 0.001) and 0.853 after adjustment (p < 0.001). For Tomita scores, the correlation coefficient was -0.994 (p < 0.001) both before and after adjustment.

**Conclusions:**

Tomita scores more accurately predicted survival than Tokuhashi scores. It is helpful to evaluate both scoring systems with adjustment for primary malignancy depending on the clinical setting. Patients with Tomita scores less than or equal to 8 and Tokuhashi scores greater than or equal to 6 are recommended to undergo surgical management.

## Background

The primary goals of surgery for spinal metastasis are generally pain relief and the preservation of ambulatory functions [1]. In the last 2 decades, the development of surgical methods has resulted in improved postoperative quality of life. However, the prognostic factors and the role of surgery in the treatment of metastatic spinal diseases remain unclear. Several authors suggested predictive systems to evaluate life expectancy, which helps in decision-making concerning the subsequent treatment strategy after the development of metastatic spinal diseases. In addition, the Tomita and Tokuhashi systems have been widely accepted in clinical settings [2,3]. However, the systems greatly differ in their use of scoring parameters and the relative importance of various preoperative factors.

This aim of this study is to determine the effectiveness to determine the effectiveness of the Tomita and Tokuhashi scoring systems in determining patient prognosis. We also evaluated the validity and predictive value of the Tomita and Tokuhashi scoring systems. We discuss several aspects to adjust both scoring systems to improve their prediction of survival. Moreover, we suggest modified criteria for selecting treatment strategies according to the adjusted scores.

## Methods

### Patient selection

The outcomes of 153 patients who underwent surgical treatment for metastatic spinal diseases between January 2006 and June 2011 were retrospectively reviewed. Patients with spinal metastasis who had undergone previous surgery, children less than 18 years old, and patients lost to follow-up were excluded from the study. Finally, 112 patients were enrolled in our study. The records of all patients were retrieved, and demographic data were collected, including age, sex, affected period, and surgical outcome. Relevant clinical data were obtained through a review of the patients’ charts and operative reports. The extent of surgical resection, use of any adjuvant therapy, length of follow-up, evidence of recurrence, and complications were also noted. The study was independently reviewed and approved by the institutional review boards of Samsung Medical Center.

### Surgery and adjuvant therapy

All patients were evaluated on plain radiographs, computed tomography (CT), and magnetic resonance imaging of the spine. Bone scintigraphy and positron emission-CT, and CT of the chest, abdomen, and brain, were also performed to evaluate systemic metastasis. Candidates for surgical management were carefully selected on the basis of the following surgical indicators: 1) more than 6 months of life expectancy as predicted by medical oncologists based on general performance and response to chemotherapy; 2) the presence of unendurable severe pain; and 3) the presence of neurologic deficits. Each patient had undergone surgical treatment with appropriate procedures depending on the surgeon’s decision. The operative procedures included excisional surgery with/without spondylectomy and palliative surgery with/without posterior fixation. The extent of surgical resection was classified into four types as follows: type 1, total *en bloc* resection; type 2, marginal or piecemeal lesionectomy; type 3, decompression with posterior fixation; and type 4, decompression without internal fixation. Adjuvant treatments including chemotherapy or radiation therapy were performed in consideration of the individual disease course. After surgery, the patients were regularly followed up every month. The length of the follow-up period was defined as the period from the date of surgery to the patient’s most recent clinic visit.

### Analysis of prognostic factors related to survival

Several preoperative variables were analyzed, including age, gender, the type of primary malignancy, the involved spinal level, and the type of operation. Other factors including the interval between the diagnosis of primary malignancy and that of spinal metastasis and the interval between the diagnosis of spinal metastasis and the operation date were also analyzed as independent prognostic variables. The interval between the diagnoses of primary malignancy and spinal metastasis was classified into 3 categories as follows: 1) metastasis already present at the time of the primary diagnosis (with no time interval), 2) early metastasis (within 1 year), and 3) late metastasis (after 1 year). An interval of less than a month between the diagnoses was considered to indicate that spinal metastasis was already present. The interval between the detection of metastasis and surgery was classified as 1) immediate surgery and 2) delayed surgery depending on whether preoperative adjuvant treatment was needed. An interval of less than a month between the detection of spinal metastasis and surgery was considered immediate surgery. The overall survival period was analyzed according to the type of primary malignancy. The histological scoring criteria of the Tomita and Tokuhashi systems were re-classified according to the survival period for each cancer type assessed in the present study.

### Analysis of the validity of the scoring systems

For preoperative prognostic scoring, the modified Tokuhashi and Tomita scoring systems were used [2,3]. For each primary malignancy, new scoring criteria were applied in each system according to the survival results of this study. Each revised scoring system was adjusted in line with the scoring principle described in the original articles. The validity of the prognostic scores was assessed using correlation analysis between survival and the scores. The mean or median survival and hazard ratio in each scoring group were calculated and compared with previously reported values.

### Statistical analyses

Student’s *t*-test was used for continuous and parametric values, and the chi-squared test and Fisher’s exact test were used for categorical dates and values, respectively. Overall survival was estimated using the Kaplan-Meier method. The log-rank test and Cox proportional hazards model were utilized to determine factor-influenced progression. Correlation studies were performed using Spearman’s rank correlation and simple regression to investigate the relationships between overall survival and the prognostic scores. Statistical analysis was conducted using commercially available software (SPSS statistics version 20.0; SPSS, Chicago, IL). A p-value < 0.05 was regarded as statistically significant.

## Results

### Patient demographics

Overall, the outcomes of 112 consecutive patients (88 males and 24 females) with a median age of 57 years (range, 28–76 years) were retrospectively reviewed. Spinal metastasis was detected at the cervical level in 20 patients, the thoracic level in 49 patients, the lumbosacral level in 27 patients, and multiple levels in 11 patients. The most common types of primary malignancy were lung, liver, and gastrointestinal cancers, which were observed in 23, 19, and 19 patients, respectively. The demographic data of the study population are summarized in Table 1.

**Table 1:** Demographic data of the study population

**Primary site**	**Age**		**Gender**		**Spinal level**				**Total**
	**≤65**	**>65**	**Male**	**Female**	**Cervical**	**Thoracic**	**Lumbo-sacral**	**Multiple**	
Lung	21	2	17	6	8	10	3	2	23
Liver	18	1	16	3	6	8	5	0	19
Rectum	6	3	6	3	1	2	6	0	9
Kidney	6	1	7	0	0	1	4	2	7
Prostate	6	0	4	2	0	3	2	1	6
Colon	3	2	3	2	1	2	2	0	5
Breast	0	5	5	0	1	2	2	0	5
MM	4	1	5	0	1	4	0	0	5
Stomach	4	0	2	2	1	2	1	0	4
Uterus	0	4	4	0	0	2	0	2	4
Head/Neck^*^	4	0	3	1	1	3	0	0	4
GB/Biliary	1	2	3	0	0	3	0	0	3
Esophagus	3	0	1	2	0	2	0	1	3
Mediastinal^†^	3	0	3	0	0	2	0	1	3
Lymphoma	3	0	3	0	0	2	1	0	3
MPNST	2	0	2	0	1	0	1	0	2
Thyroid	0	2	1	1	0	1	0	1	2
Bladder	0	1	0	1	0	1	0	0	1
Others^‡^	3	1	2	2	0	3	0	1	4
Total	87	25	87	25	21	53	27	11	112

Results are presented as number of patients.

^*^Nasopharyngeal cancer, glottic cancer, soft palate cancer; ^†^thymoma, mesothelioma; ^‡^pancreatic cancer, urethral cancer, adrenal cancer, melanoma. MM, multiple myeloma; GB, gall bladder; MPNST, malignant peripheral nerve sheath tumor.

### Surgical results

En bloc resection (type 1) of tumors was achieved in 3 patients, and marginal or intralesional resection (type 2) was performed in 56 patients. Palliative surgery was performed in 53 patients, including 41 patients who underwent decompression with fixation (type 3) and 12 patients who underwent decompression without fixation (type 4). In our series, the 30-day morbidity rate was 16.1% (16 cases), and the 30-day mortality rate was 4.5% (5 cases). Surgical complications involved pneumonia in 6 patients, wound infection/dehiscence in 5 patients, and postoperative hematoma, deep vein thrombosis, and cerebrospinal fluid leakage in 2 patients. Other complications included urinary tract infection, gastrointestinal bleeding, and screw loosening or malposition in 3 patients. Life-threatening complications, such as sepsis, acute respiratory failure, myocardial infarction, pulmonary embolism, and intracranial hemorrhage, were also present in 5 patients. Four patients (3.6%) experienced surgical complications requiring a second surgery, such as wound dehiscence, postoperative bleeding, and cerebrospinal fluid leakage. The surgical results of various primary malignancies, including postoperative complication, mortality, and the 1-year survival rate, are summarized in Table 2.

**Table 2:** Outcomes of the surgical candidates

**Primary malignancy**	**Surgical treatment**				**Morbidity (%)**	**Mortality (%)**	1YSR*,%	**Follow up, months†**
	**Excisional Op**		**Palliative Op**					
	**Type 1**	**Type 2**	**Type 3**	**Type 4**				
Lung	0	9	11	3	8.7	8.7	13.0	4.3 (0.7 - 20.1)
Liver	0	10	5	4	10.5	5.3	31.6	8.5 (0.6 - 45.7)
Rectum	0	7	2	0	11.1	0	0	3.3 (1.0 - 4.8)
Kidney	0	4	3	0	0	0	14.3	9.2 (5.0 - 26.2)
Prostate	0	1	5	0	50.0	0	16.7	5.4 (3.9 - 36.7)
Colon	0	3	2	0	20.0	0	80	33.4 (5.6 - 46.2)
Breast	1	4	0	0	0	0	0	4.7 (4.5 - 10.4)
MM^‡^	0	4	1	0	0	0	100	27.8 (17.5 - 53.7)
Stomach	0	3	1	0	0	0	0	4.3 (2.7 - 10.5)
Uterus	1	2	1	0	75	0	25.0	7.6 (2.1 - 40.4)
Head/Neck	1	2	1	0	50	0	50.0	26.8 (3.9 - 48.5)
GB/Biliary	0	1	1	1	66.7	33.3	0	4.5 (0.7 - 5.3)
Esophagus	0	2	1	0	33.3	0	33.3	2.3 (2.1 - 13.5)
Mediastinal	0	0	2	1	0	33.3	33.3	6.3 (0.5 - 32.3)
Lymphoma	0	1	2	0	33.3	0	66.7	60.9 (11.6 - 75.1)
MPNST	0	2	0	0	0	0	50.0	24.5 (3.1 - 45.9)
Thyroid	0	0	1	1	0	0	100	45.9 (17.1 - 74.7)
Bladder	0	0	0	1	0	0	0	2.7
Others	0	1	2	1	0	0	25.0	6.8 (2.0 - 16.5)
Total	3	56	41	12	16.1	4.5	27.7	5.9 (0.5 - 75.1)

^*^1-year survival rate; ^†^median follow-up period with ranges; MM, multiple myeloma; GB, gall bladder; MPNST, malignant peripheral nerve sheath tumor.

### Prognostic value of preoperative factors

All 112 candidates were evaluated for follow-up analysis. The 1-year survival rate was 26.8%. The last evaluation was performed in June 2012, producing a median follow-up time of 5.8 months after the operation (range, 0.5–75.1 months). Ninety-nine patients died during the follow-up period. The most common causes of death were major organ failure due to multiple metastases. Two patients died because of septic shock and another 2 patients because of myocardial infarction and pulmonary embolism, respectively. 1 patient died because of brain metastasis.

In univariate analysis, age (p = 0.235), gender (p = 0.460), the involved spinal level (p = 0.373), and the type of surgical methods (p = 0.228) had no significant relationship with survival (Table 3). However, the interval from the diagnosis of primary malignancy to that of spinal metastasis (p = 0.023) and the interval from the diagnosis of spinal metastasis to surgery (p = 0.039) were significantly related to survival. The survival period after surgery was longer in patients in whom spinal metastasis was present at the time of primary malignancy diagnosis and in those with late metastasis than in those with early metastasis. Concerning the timing of surgery, immediate surgery within a month was associated with a better prognosis than delayed surgery.

**Table 3:** Survival analysis of each preoperative factor

**Preoperative factor**		**No. of patients**	**Survival period (months)**		**P value**
			**Mean (95% CI)**	**Median (95% CI)**	
Age	Less than 65	87	19.4 (13.6 - 25.2)	5.8 (4.3 - 7.3)	0.235
	65 or more	25	8.6 (5.7 - 11.6)	6.1 (3.8 - 8.5)	
Gender	Male	87	16.5 (11.3 - 21.7)	5.8 (4.9 - 6.8)	0.460
	Female	25	20.7 (9.9 - 31.4)	8.0 (2.0 - 13.9)	
Spinal level	Cervical	21	9.9 (3.6 - 16.2)	4.2 (3.7 - 4.6)	0.373
	Thoracic	53	17.7 (11.1 - 24.3)	6.5 (3.4 - 9.7)	
	Lumbosacral	27	14.9 (8.5 - 21.3)	5.9 (4.9 - 6.8)	
	Multiple	11	18.3 (2.4 - 34.1)	7.7 (3.4 - 11.9)	
Operation type	Type 1	3	35.7 (16.2 - 55.3)	N/A	0.228
	Type 2	56	14.1 (9.7 - 18.6)	5.6 (2.2 - 9.0)	
	Type 3	41	17.6 (9.6 - 25.7)	6.1 (5.3 - 6.9)	
	Type 4	12	8.8 (2.1 - 15.6)	5.3 (0.2 - 10.3)	
Time interval to metastasis^*^	None	36	24.4 (14.7 - 34.1)	7.8 (1.9 - 13.8)	0.023*
	<1 year	20	7.4 (2.9 - 11.9)	5.1 (2.0 - 8.2)	
	>1 year	56	17.3 (10.7 - 24.0)	5.5 (4.3 - 6.8)	
Time interval to operation^†^	Immediate	84	19.7 (13.9 - 25.5)	6.2 (3.8 - 8.7)	0.039^†^
	Delayed	28	8.1 (4.5 - 11.7)	5.0 (3.0 - 7.1)	
Total		112	17.3 (12.6 - 22.1)	5.8 (5.0 - 6.7)	

^*^The interval between the diagnosis of primary malignancy and that of spinal metastasis; ^†^the interval between the diagnosis of spinal metastasis and surgery: these intervals were significantly associated with survival according to the log-rank test. N/A, estimation data not available due to the lack of uncensored cases.

The mean survival of the 112 patients according to the type of primary malignancy is summarized in Table 4. Primary malignancy was classified as favorable, intermediate, or poor according to the mean survival period. Lymphoma, multiple myeloma, thyroid cancer, and breast cancer had favorable prognoses with a mean survival of 54.4 months (95% confidence interval [CI] = 39.5–69.2 months). Cancers of the uterus, liver, prostate, and kidney and other unspecified malignancies had an intermediate prognosis with a mean survival of 14.7 months (95% CI = 10.1–19.4 months). Lung, gastrointestinal, hepatobiliary, and bladder cancers had a poor prognosis with a mean survival of 5.2 months (95% CI = 3.9–6.4 months). The hazard ratios of the favorable, intermediate, and poor prognosis groups were 1, 5.21, and 12.86, respectively.

**Table 4:** Scoring based on the primary malignancy

**Primary malignancy**	**No.**	**Survival period (months)**		**Tokuhashi score**		**Tomita score**		**Prognosis**
		**Mean (95% CI)**	**Median (95% CI)**	**Original**	**Adjusted**	**Original**	**Adjusted**	
Lymphoma	3	53.9 (20.1 - 87.8)	-	-	5	-	1	Favor
MM^*^	5	46.5 (33.8 - 59.2)	-	-	5	-	1	
Thyroid	2	45.9 (5.9 - 85.9)	17.1	5	5	1	1	
Breast	5	31.4 (15.4 - 47.4)	-	5	5	1	1	
Uterus	4	14.4 (0.0 - 29.5)	3.9 (0.0 - 12.9)	3	4	2	2	Intermediate
Liver	19	12.2 (6.6 - 17.7)	8.5 (5.6 - 11.5)	1	4	4	2	
Prostate	6	10.9 (1.6 - 20.2)	5.1 (4.1 - 6.0)	5	3	1	2	
Kidney	7	10.8 (5.9 - 15.7)	9.2 (5.2 - 13.3)	3	3	2	2	
Lung	23	6.0 (3.7 - 8.3)	4.3 (1.3 - 7.3)	0	1	4	4	Poor
Esophagus	3	6.0 (0.0 - 13.4)	2.3 (2.0 - 2.6)	0	1	-	4	
Colon	5	6.0 (3.8 - 8.2)	4.7 (4.5 - 4.9)	2	1	4	4	
Stomach	4	5.5 (2.0 - 8.9)	3.4 (0.8 - 5.9)	0	0	-	4	
GB/Biliary	3	3.5 (0.7 - 6.3)	4.5 (0.0 - 10.7)	1	0	-	4	
Rectum	9	3.1 (2.1 - 4.0)	3.3 (2.4 - 4.3)	4	0	-	4	
Bladder	1	2.7	2.7	0	0	-	4	
Others^*^	13	18.9 (7.3 - 30.4)	6.3 (0.0 - 14.3)	2	2	-	2	Intermediate
Overall	112	17.3 (12.6 - 22.1)	5.8 (5.0 - 6.7)					

Primary malignancy was categorized according to the Tokuhashi score (0 to 5) or Tomita score (1, 2, or 4). The scores for certain primary malignancies, including uterine, liver, and prostate cancers, were reassigned for adjustment. The scores for colon and rectal cancers in the Tokuhashi system were reassigned to indicate a worse prognosis in line with the Tomita system. ^*^Other types of primary malignancy: head and neck region, mediastinum, and other malignancies, that is, pancreatic cancer, urethral cancer, adrenal cancer, and melanoma. MM, multiple myeloma; GB, gall bladder; MPNST, malignant peripheral nerve sheath tumor. Dash means no value for parameter.

### Validity of the scoring systems

**Figure 1:**
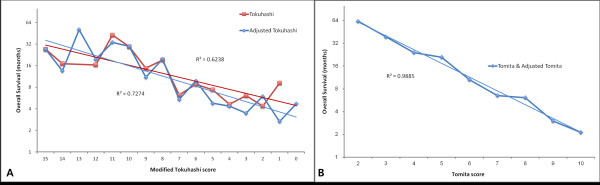
**Simple linear regression of two scoring systems. (A)** Tokuhashi scores and mean survival. The coefficient of determination (*R*^2^) was 0.6238 (red line) before adjustment and 0.7274 (blue line) after adjustment. **(B)** Tomita scores and mean survival. *R*^2^ according to simple regression analysis was 0.9885. There was no difference between Tomita scores before and after adjustment.

The predictive value of the Tokuhashi and Tomita scores was analyzed with/without adjustment for the primary malignancy. For Tokuhashi scores, each scoring system was linearly correlated with survival on a logarithmic scale (Figure 1A). The correlation coefficient was 0.790 before adjustment (p = 0.001) and 0.853 after adjustment (p < 0.001). In simple regression analysis, the coefficient of determination (R^2^) was 0.624 before adjustment (p = 0.001) and 0.727 after adjustment (p < 0.001). After adjustment, the Tokuhashi scores were more strongly correlated with survival than those before adjustment. On the contrary, the mean survival predicted by each Tomita score displayed no difference between before and after adjustment. For Tomita scores, the correlation coefficient on a logarithmic scale was -0.994 (p < 0.001), which indicated a stronger correlation than the original and adjusted Tokuhashi scores (Figure 1B). R^2^ as determined by simple regression analysis was 0.989 (p < 0.001).

### Predictive value of the scoring groups

**Figure 2:**
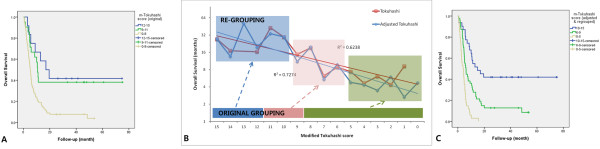
**Estimated survival curves of the Tokuhashi scoring systems. A)** Overall survival for the original Tokuhashi scoring system. **B)** Regrouping into score groups of 0–5, 6–9, and 10–15 based on the survival data. **C)** Overall survival for the adjusted and regrouped scoring systems.

**Figure 3:**
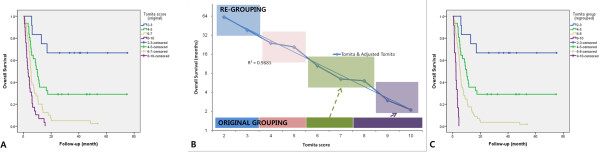
**Estimated survival curves of the Tomita scoring systems. A)** Overall survival for the original Tomita scoring system. **B)** Regrouping into score groups of 2–3, 4–5, 6–8, and 9–10 based on the survival data. **C)** Overall survival for the regrouped Tomita scoring system.

Patients were classified into prognostic groups according to their Tokuhashi and Tomita scores (Table 5). Tokuhashi scores were grouped as follows: 0–8, 9–11 and 12–15. The mean survival times for these groups were 37.1, 32.8 and 9.4 months, respectively, before adjustment (Figure 2A), and 38.3, 26.9 and 9.4 months, respectively, after adjustment. However, the similarity of survival between before and after adjustment was improved after adjustment upon reclassification of the scores into groups of 0–5, 6–9 and 10–15 (Figure 2B). For the reclassified Tokuhashi scoring groups, the mean survival times were 36.5, 12.9, and 4.4 months, respectively, for the 3 groups (Figure 2C). In Spearman’s correlation analysis, the correlation between the estimated survival and the scoring groups was insignificant for group 2 (p > 0.050), but this score became significant after regrouping (p = 0.001). The hazard ratios for the adjusted and regrouped Tokuhashi scoring groups were 1, 2.42, and 6.17, respectively.Tomita scores were grouped as follows: 2–3, 4–5, 6–7, and 8–10. The mean survival ties for these 4 groups were 53.6, 27.0, 9.0, and 4.6 months, respectively (Figure 3A). Tomita scores were also regrouped into ranges of 2–3, 4–5, 6–8, and 9–10 based on the mean survival (Figure 3B). After regrouping, the mean survival times were 8.4 months for the 6–8 score group and 2.6 months for the 9–10 score group (Figure 3C). The hazard ratios for the 4 groups were 1, 3.30, 6.96, and 12.65, respectively, before regrouping and 1, 3.33, 7.65, and 42.62, respectively, after regrouping.

**Table 5:** Comparison of the validity of Tokuhashi and Tomita scores

**Survival groups**			**Scores**	**No. of cases**	**Survival period**		**Hazard ratio**	**Sig**^**†**^
					**Mean (95% CI)**	**Median (95% CI)**		
Tokuhashi	Original	1	12-15	12	37.1 (18.9 - 55.3)	17.1 (5.9 - 28.2)	-	0.000
		2	9-11	21	32.8 (18.6 - 47.0)	10.7 (9.1 - 12.3)	1.23	0.654
		3	0-8	79	9.4 (6.6 - 12.3)	5.3 (4.4 - 6.1)	3.17	0.004
	Adjusted	1	12-15	14	38.3 (21.6 - 54.9)	17.1 (5.0 - 29.1)	-	0.000
		2	9-11	27	26.9 (15.1 - 38.8)	8.9 (3.0 - 14.8)	1.67	0.222
		3	0-8	71	9.0 (6.1 - 11.9)	4.9 (4.1 - 5.6)	3.56	0.001
	Regrouped	1^*^	10-15	31	36.5 (24.9 - 48.1)	14.9 (5.3 - 24.5)	-	0.000
		2^*^	6-9	47	12.9 (8.4 - 17.3)	6.2 (4.6 - 7.8)	2.42	0.002
		3^*^	0-5	34	4.4 (3.3 - 5.5)	3.9 (2.1 - 5.8)	6.17	0.000
Tomita	Original	1	2-3	12	53.6 (36.3 - 70.8)	N/A	-	0.000
		2	4-5	31	27.0 (16.2 - 37.8)	10.0 (7.7 - 12.4)	3.30	0.028
		3	6-7	40	9.0 (5.7 - 12.4)	5.3 (4.4 - 6.1)	6.96	0.000
		4	8-10	29	4.6 (3.2 - 6.0)	3.3 (2.2 - 4.5)	12.65	0.000
	Regrouped	1^*^	2-3	12	53.6 (36.3 - 70.8)	N/A	-	0.000
		2^*^	4-5	31	27.0 (16.2 - 37.8)	10.0 (7.7 - 12.4)	3.33	0.027
		3^*^	6-8	57	8.2 (5.7 - 10.6)	5.5 (4.5 - 6.5)	7.65	0.000
		4^*^	9-10	12	2.4 (1.7 - 3.1)	2.3 (1.7 - 2.9)	42.62	0.000

^*^Reclassified groups after reorganization of prognostic scores. ^†^*P*-value obtained by Cox regression; *P* <0.050 denotes statistical significance (Sig) N/A, estimation data not available due to the lack of uncensored cases. Dash means no value for parameter.

## Discussion

In this study, several preoperative variables were examined to identify their prognostic value. According to our results, gender and age have no significant relationship with survival, which is consistent with previous studies. However, it has been reported that patients younger than 65 years have a significantly high level of satisfaction after surgical management [1]. The interval between the primary diagnosis and the diagnosis of spinal metastasis was presently identified as an independent prognostic factor (p = 0.042). This interval might be also relevant to cancer progression, which can be easily assessed regardless of other clinical findings. The timing of the operation was also significantly relevant to survival (p = 0.027), which implies that the role of surgery in the treatment of metastatic spinal diseases is not limited to improvement of a patient’s quality of life, but it may also be related to life expectancy.

Although various scoring systems have been described in previous studies, a few selected systems have been widely used and recommended by the Global Spinal Tumor Study Group [4]. Tokuhashi et al. designed a scoring system to predict the prognosis of patients with metastatic spinal diseases and select the most appropriate treatment strategy [2]. Tomita et al., who implied that the Tokuhashi scoring system does not consider the relative importance of individual prognostic factors, suggested another scoring system that focused on surgical strategies [3]. The Tomita scoring system consists of only 3 prognostic factors, namely the grade of the primary malignancy, presence of visceral metastases, and presence of bone metastases.

In both scoring systems, the type or grade of the primary malignancy is one of the most important factors affecting survival. Tokuhashi et al. assigned 0 points for a mean survival of less than 6 months, 1 point for a mean survival of approximately 6 months, 2 points for an unspecified mean survival, 3 points for a mean survival of less than 12 months, and 4 or 5 points for a mean survival of more than 12 months [3]. The Tomita score of 1, 2, and 4 for primary malignancies were based on hazard ratios obtained using Cox regression analysis (1, 1.82, and 4.08, respectively, in the original article) [1]. However, the survival results for each cancer group were not consistent between those studies. For example, rectal cancer was categorized into the mild prognostic group in the Tokuhashi system, whereas it was classified as a high-grade malignancy in the Tomita system. The scoring of primary malignancy in the Tokuhashi system was also inconsistent with the survival data from other studies [5].

In our study, the survival results for each malignancy were more consistent with the Tomita system. We reclassified the scoring of primary malignancy based on our results; i.e., colorectal cancer was given a score of 0 points instead of 2–4 points, and liver cancer was given a score of 4 points instead of 1 point. As the prognosis and disease course vary widely depending on the characteristics of the primary malignancy, several recent studies discussed prognostic scoring focused on individual cancer groups [6,7]. Recently, the development of new cancer therapies places the scoring systems in a new scenario, implying a need for reassessment of its current usefulness. Recent studies have shown both favorable and unfavorable results in this paradigm. Accordingly, the scoring for primary malignancy should be carefully evaluated in consideration of each institute’s clinical settings.

Comparing the validity of the 2 scoring systems is not a simple task. Several institutes reported their clinical results using either the Tomita or Tokuhashi scoring system [8-11]. Some authors attempted to compare the accuracy rate of the Tomita or Tokuhashi scoring system in series of patients treated with earlier cancer protocols, Padalkar et al. compared the usefulness of the two scoring system in a prospective series of 102 patients undergoing surgery for spinal metastasis. Type of primary tumor was not found to be significantly associated with survival according to the revised Tokuhashi scoring system (P = .9131,). Stepwise logistic regression revealed that the Tomita score correlated more closely with survival than the Tokuhashi score. They suggested that Tomita scores had a stronger correlation with survival than Tokuhashi scores [5]. Conversely, Ulmar et al. reported that Tokuhashi scores appear to be more valuable than Tomita scores in survival analysis of 37 consecutive patients with spinal metastases secondary to renal cancer. [12]. Zou et al. reported that Tokuhashi scores more accurately predicted short-term survival, whereas Tomita scores were more useful for predicting long-term survival [13].

Hessler et al. evaluated the reliability of the Tokuhashi scoring system in patients with lung cancer. The authors concluded that the Tokuhashi scoring system is not an optimal tool for decision-making in patients with spinal metastasis of lung cancer, and they mentioned a risk of undertreatment in certain patients with unfavorable scores who ultimately survived longer than 12 months [14]. In our study, Tomita scores had a stronger correlation with mean survival (R^2^ = 0.9885). Although Tokuhashi scores had a weaker correlation with mean survival, the correlation was improved after adjustment of the scoring based on the primary malignancy (R^2^ = 0.6238–0.7274). Therefore, we suggest that the Tomita system is a simpler and more accurate tool in preoperative evaluation for predicting life expectancy.

**Figure 4:**
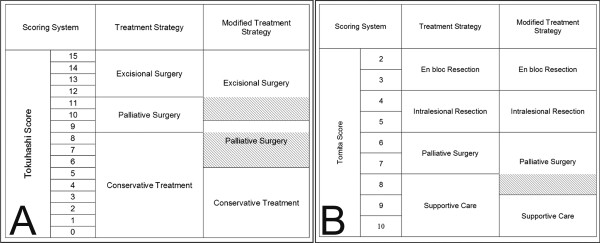
**Proposed treatment strategy according to the A) Tokuhashi and B) Tomita score systems.** Tokuhashi scores of 10–11 were reclassified to recommend excisional surgery (either en bloc or intralesional resection) instead of palliative surgery (oblique lines). A Tomita score of 8 and Tokuhashi scores of 10–11 have been reclassified to recommend palliative surgery instead of conservative treatment (oblique lines).

Another important issue in planning treatment strategies is deciding between surgical management and supportive care. It is generally accepted that surgical management should be considered for patients with a life expectancy of at least 3 months [4]. Tokuhashi et al. predicted life expectancy by grouping their scores, resulting in scores of 0–8 for a mean survival of less than 6 months, 9–11 for a mean survival of 6–12, and 12–15 for a mean survival exceeding 12 months [3]. According to Tokuhashi et al., surgical management is not recommended for patients with Tokuhashi scores of 0–8, as their life expectancy is less than 6 months. Tomita et al. also grouped their scores, resulting in scores of 2–3 for a mean survival of 49.9 months, 4–5 for a mean survival of 23.5 months, 6–7 for a mean survival of 15.0 months, and 8–10 for a mean survival of 5.9 months [2]. They recommended that patients with Tomita scores of 8–10 should receive supportive care. However, our results illustrated that more patients can be considered for surgical management than identified by both scoring systems. Our data indicated that patients with a Tomita score of 8 have an estimated survival exceeding 6 months, which was obviously different from the estimated survival of patients with scores of more than 9. We suggest that a Tomita score of 8 should be reclassified into the upper group, in which patients are recommended to consider palliative surgery rather than supportive care. Recently, several authors reported that the Tokuhashi system was not highly accurate for predicting survival in patients with spinal metastasis, treated or not surgically, and it was particularly imprecise in patients with an intermediate score (9–11 points) [6,15]. In our study, because scores of 10–11 were associated with a mean survival exceeding 12 months in the Tokuhashi system, these scores were reclassified to recommend excisional surgery (either en bloc or intralesional resection) instead of palliative surgery. Furthermore, patients with Tokuhashi scores of 6–8 had a life expectancy exceeding 6 months, and they should be reclassified into the next survival category recommending more aggressive treatment (Figure 4).

## Conclusions

In conclusion, the interval between the diagnosis of the primary malignancy and that of spinal metastasis and the interval between the diagnosis of spinal metastasis and surgery were identified as independent prognostic factors that are practically simple to assess. Concerning the scoring systems, Tomita scores more accurately predicted survival than Tokuhashi scores. We also suggest that patients with Tomita scores of 8 or less and Tokuhashi scores of 6 or more should be considered for surgical management rather than conservative treatment.

## Abbreviations

CT: computed tomography.

## Competing interests

The authors declare that they have no competing interests.

## Authors’ contributions

JK collected data and drafted the manuscript. SHL made substantial contributions to the conception and design of the study, as well as data collection, and revised the manuscript critically for important intellectual content. SJP, ESK, and SSC participated in the analysis and interpretation of data, WE, CSL participated in study design. All authors read and approved the final manuscript.
